# Current knowledge on *Inquilinus limosus,* a scarcely researched human pathogen

**DOI:** 10.1186/s12866-024-03617-6

**Published:** 2024-11-13

**Authors:** Oluwafemi M. Akinnurun, Thomas Riedel, Stephanie Müller, Boyke Bunk, Percy Schröttner

**Affiliations:** 1 Institute for Medical Microbiology and Virology, Faculty of Medicine and University Hospital Carl Gustav Carus, Technische Universität Dresden, Dresden, Germany; 2 Leibniz Institute DSMZ-German Collection of Microorganisms and Cell Cultures, Brunswick, Germany; 3 German Center for Infection Research (DZIF), Partner Site Hannover-Braunschweig, Brunswick, Germany; 4 Department of Medicine I, University Hospital Carl Gustav Carus, Technische Universität Dresden, Dresden, Germany; 5 Institute for Clinical Chemistry and Laboratory Medicine, Faculty of Medicine and University Hospital Carl Gustav Carus, Technische Universität Dresden, Dresden, Germany

**Keywords:** *Inquilinus limosus*, Cystic fibrosis, Rare bacterial pathogen, Underestimated bacterial pathogen

## Abstract

*Inquilinus limosus* belongs to the class of the Alphaproteobacteria and was first described in 2002. So far, the species has mainly been isolated from respiratory specimens of patients with cystic fibrosis. A main characteristic of *Inquilinus limosus* is the prolonged time until bacterial colony growth is detectable. As the defined incubation times in many laboratories are too short to detect the growth of *Inquilinus limosus*, it is likely that the species is less frequently detected in the clinical setting than it actually occurs. This also explains why there are currently only very few data on the incidence available. Furthermore, as an uncommon pathogen, *Inquilinus limosus* may be familiar to only a few specialised clinicians. Due to these reasons, only little research (e.g. case reports and research papers) have been published on this species to date. However, given that a clear human pathogenic significance can be deduced from the existing literature, we have decided to present the current state of knowledge in this review and to address further aspects for the future elucidation of the pathogenesis of *Inquilinus limosus*.

## Introduction

The bacterial genus *Inquilinus* belongs to the class of Alphaproteobacteria and is part of the *Azospirillaceae* family [1]. According to the „List of Prokaryotic names with Standing in Nomenclature" (LPSN), there are currently two validly published names assigned to the genus *Inquilinus* [1]. The type species, *I.* *limosus*, was isolated from the respiratory secretions of a patient with cystic fibrosis (CF) in 1998 and described in 2002 [2]. The second species, *I. ginsengisoli*, was obtained from soil samples and described by Jung et al. in 2011 [3].

In the course of the description of *I. limosus* by Coenye et al., another isolate (AU1979) was found, which, however, showed only a 53% match in DNA-DNA hybridisation compared to the type strain of *I. limosus* [2]. Since this value is below the 70% cut-off (which defines the species level), it is very likely that this isolate represents another yet undefined species of the genus *Inquilinus* [2, 4]. However, since the sequences of the 16S rRNA gene show a strong homology between the *I. limosus* type strain (GenBank accession no. AY043374) and the strain AU1979 (GenBank accession no. AY043375), it can be assumed that both strains belong to the genus *Inquilinus*. Furthermore, another strain (DS15158; previously isolated by Pitulle et al.) was also investigated by Coenye et al. [2, 5]. Since in this case there are also strong homologies between the sequence of the 16S rRNA gene (GenBank accession no. AF085496) to the *I. limosus* type strain, it is also very likely that this isolate belongs to the genus *Inqulinus* as well [2]. Unfortunately, no DNA-DNA hybridisation results are currently available and therefore a clear statement can not be made, whether it is another species of the genus *Inquilinus* or even belongs to the species *I. limosus.*

While *I. limosus* was repeatedly isolated from clinical samples in recent years, there is currently no evidence of clinical significance for *I. ginsengisoli*. In this review, we therefore provide an overview on *I. limosus* and focus on the microbiological description of the species, current genomic information, options for species identification in the routine diagnostics, clinical features and pathogenicity, antimicrobial susceptibility and possibilities for antimicrobial treatment.

### Search strategy and selection of articles

A literature search was conducted in PubMed. The following keywords were used: "*Inquilinus limosus*", "*Inquilinus limosus* AND infection" and "*Inquilinus limosus* AND human". All studies published in PubMed since the initial description up to 1st of April 2023 were considered. Only English-language literature was included. All available manuscripts (including the first description and clinical case reports associated with *I. limosus*) were evaluated for their relevance in the preparation of this review.

### Type strain and genomic data

The type strain (AU476^T^) is currently deposited in five culture collections, "The German Collection of Microorganisms and Cell Cultures" in Braunschweig (DSM 16000^ T^), the "Culture Collection University of Gothenborg" (CCUG 45653^ T^), the Master Collection of the Institute Pasteur, France (CIP 108342^ T^), the of the Czech culture collection (CCM 7171^ T^) and the "BCCM/LMG Bacteria Collection of Ghent University" (LMG 20952^ T^). Four genomes are currently available in the GeneBank, the genome for the type strain DSM 16000^ T^ (GCA_000423185.1) and three other genomes derived from bioprojects (GCA_002195995.1, GCA_000767795.1, GCA_019508225.1) [6]. A summary of the relevant genome information is provided in Table 1.

**Table 1:** Overview of the available genome sequences of *Inquilinus limosus* (GeneBank)

Strain name of sub-species classificartion	Total sequence length [Mb]	GC% content in %	Protein coding genes	Sequencing technology	GenBank assembly accession	Source
DSM 16000^ T^	7.41	69.9	6952	Illumina HiSeq 2000	GCA_000423185.1	clinical sample (CF patient)
Inq	7.69	69.9	7.098	Illumina HiSeq 1500	GCA_002195995.1	contaminated soil
MP06	6.93	69.6	6938	454	GCA_000767795.1	clinical sample (CF patient)
YM_69_17	5.93	69.9	5877	Illumina HiSeq	GCA_019508225.1	n.g

*n.g*. not given

### Phenotypic characteristics of *Inquilinus limosus*

The bacteria is a gramnegative rod with a width between 1.5 and 2 µm and a length of 3.5 µm [2, 5]. Colonies of *I. limosus* usually grow mucoidal and slowly [2, 5]. The optimal growth temperatures are indicated between 35 °C and 42 °C, but growth is still possible at 25 °C [2, 5]. *I. limosus* is not motile [2, 5]. The species is able to utilise a number of carbohydrate sources, which include D-fructose, L-fucose, D-galactose, maltose, D-mannose, D-raffinose or D-sorbitol, but not D-glucose [2, 5]. For this reason, it is counted among the so-called „non-fermenters “. Furthermore, mannitol, sorbitol, inositol, rhamnose, sucrose, melibiose, amygdalin, arabinose and citrate are not utilised [2]. Although the type strain does not grow in 6% NaCl, other isolates of the species are able to tolerate this high NaCl concentration [2]. Therefore, a variable NaCl tolerance can be assumed. *I. limosus* expresses oxidase, catalase, phosphatase, urease, gelatinase, proline aminopeptidase, lipase, and phosphatase [2]. *I. limosus* is not able to produce H_2_S, indole and acetoin [2].

### Epidemiology

Most *I. limosus* isolates have been obtained from clinical samples. However, to the best of our knowledge, one isolate has been obtained from soil samples (strain „Inq“, see Table 1). Since, the closely related species *I. ginsengisoli* was detected in soil as well, it can be assumed that *I. limosus* may also be found in environmental samples [3, 7]. In a recent study, *I. limosus* was isolated from the oral cavity of a domestic cat [8]. However, it is unclear how or if the cat may have also acquired the bacteria from environmental sources. There are currently two studies that report incidences of *I. limosus*. In both studies, the focus was on the cultural detection of the species. While Wellinghausen assumes an incidence of 0.9%, Bittar et al. suggested an incidence of 2.8% [7, 9]. However, these data must be evaluated with caution, as they refer to the patient population of both institutions and therefore cannot be regarded as generally valid. In order to determine reliable incidences, it is therefore necessary to conduct a broad multicentre study with clearly defined study criteria. In addition, it would be important for such an approach to specify suitable conditions for bacterial cultivation (e.g. growth of *I. limosus* is possible on Columbia Blood Agar, Chocolate Agar and *Burkholderia cepacia* selective agar but not always on MacConkey Agar), a sufficient incubation period (at least 72 h) and suitable methods for species identification.

Another possibility to approach this question could be serological and molecular investigations. In a study to examine the serum antibody response to *Inquilinus* bacterial antigen (via immunoblot) in six *Inquilinus*-positive CF patients, the results showed the presence of specific *Inquilinus* serum antibodies (IgG) in the *Inquilinus*-positive patients as compared to either healthy patients or CF patients colonized with *P. aeruginosa* [10]. In addition, the authors detected specific *Inquilinus* serum IgG antibodies in a patient one year prior to *Inquilinus* identification in the patient’s sputum [10]. In another study, *I. limosus* screening real-time PCR was done using sputum from CF patients [9]. The results showed a 100% correlation with culture positive sputum. In one of the patients, *Inquilinus* was even detected three months before the culture was positive using the real-time PCR [9]. This additionally could suggest a future role for serologic and molecular biologic diagnostics in the detection of *Inquilinus* infections. However, much work is still needed for example, to isolate and characterize the secreted proteins in the antigen–antibody reaction during *Inquilinus* infections and also in the validation and standardization of the real-time PCR. Another option to clarify this question is NGS-based amplicon analysis to investigate the microbiome of cystic fibrosis patients [11]. There are currently no data on the prevalence of *I. limosus* infections. Schmoldt et al., however, are of the opinion that the prevalence is underestimated [10].

### Identification of *Inquilinus limosus* in routine diagnostics

The identification of *I. limosus* in the course of routine diagnostics is challenging. The majority of respiratory specimens analysed from the reported case reports were sputum samples (Tables 2 and 3). However, most CF patients especially children either do not routinely produce sputum or have difficulties producing them [12, 13] thus, leading to alternative methods of sample collection such as oropharyngeal swaps or cough swaps in this patient group which in turn leads to an inaccurate diagnosis of bacteria colonisation and poses a limitation to the proper identification of the bacteria [14]. The bacteria grows well on Columbia blood, Chocolate and *Burkholderia cepacia* selective agar, but growth on MacConkey agar is not always successful [7, 15–17]. In addition, the bacteria grows slowly [7, 18]. These characteristics must therefore be considered by routine diagnostic laboratories for the detection of *I. limosus*.

**Table 2:** Examples of *Inquilinus limosus* growth and incubation times

Report	Sample	Growth medium	Incubation Time
Wellinghausen et al. [7]	Sputum	Burkholderia cepacia complex selective agar	6 days
Wellinghausen et al. [7]	Sputum	Burkholderia cepacia complex selective agar	5 days
Kiratisin et al. [16]	Blood cultures	5% sheep blood agar	Blood cultures: 3—4 days Subculture medium: 48 h
Cooke et al. [19]	Sputum	Burkholderia cepacia selective agar	Not given
Hayes Jr. et al. [20]	Sputum	OFPBL media with polymyxin B, bacitracin and lactose	Not given
Salvador-García et al. [21]	Sputum	Burkholderia cepacia complex selective agar	7 days
Cicatiello et al. [18]	Sputum	Burkholderia cepacia selective agar	5 days
Goeman et al. [22]	Blood culture	Subculture medium not given	Blood culture: 5 days Subculture medium: not given
McHugh et al. [17]	Bronchoalveolar lavage	Chocolate agar, 5% sheep blood in tryptic soy agar, MacConkey agar Subculture medium: Burkholderia cepacia selective agar	Subculture medium: 48—72 h
Ríos-López et al. [23]	Sputum	Blood, Muller-Hinton and EMB Agar	Not given

**Table 3:** Clinical features of documented *Inquilinus* cases

	**Reference**	**Age at first Inquilinus isolation/Sex**	**Material investigated**	**Clinical presentation**	**Underlying disease**	**Identification**	**Morphology**
**1**	Pitulle et al. [5]	22/F	Lung explants, BAL	Postoperative pneumonic infiltrates	CF with end-stage lung disease, bilateral living-donor lobar lung transplantation	Culture (*First description*) BIOLOG GN MicroPlate assay (*closest match was Agrobacterium radiobacter with borderline acceptability*) Whole-cell cellular fatty acid analysis (*no match*) API 20E system (*no positive reaction after 48 h*) BBL Crystal Enteric/Nonfermenter ID (*no match*) RapID NF Plus system (*no match*) 16S rRNA sequencing (*Strain DS15158*)	Very mucoid
**2**	Chiron et al. [15]	12/M	Sputum	Clinically stable	CF	API 20 NE (*Sphingomonas paucimobilis*) API 32 GN (*Pseudomonas fluorescens*) 16S rRNA sequencing (*Inquilinus limosus*)	Mucoid
**3**	Chiron et al. [15]	13/F	Sputum	Exacerbation	CF with advanced respiratory disease	API 20 NE (*Sphingomonas paucimobilis*) API 32 GN (*Undetermined*) 16S rRNA sequencing (*Inquilinus limosus*)	Mucoid
**4**	Chiron et al. [15]	8/M	Sputum	Clinically stable	CF with mild respiratory disease	API 20 NE (*Sphingomonas paucimobilis*) API 32 GN (*Undetermined*) 16S rRNA sequencing (*Inquilinus limosus*)	Non-mucoid
**5**	Chiron et al. [15]	10/M	Sputum	Clinically stable but followed by spirometric deterioration	CF with mild respiratory disease	API 20 NE (*Sphingomonas paucimobilis*) API 32 GN (*Undetermined*) 16S rRNA sequencing (*Inquilinus limosus*)	Mucoid
**6**	Chiron et al. [15]	18/M	Sputum	Exacerbation	CF with advanced respiratory disease, allergic bronchopulmonary aspergillosis	API 20 NE (*Agrobacterium radiobacter*) API 32 GN (*Klebsiella spp.*) 16S rRNA sequencing (*Inquilinus limosus*)	Mucoid
**7**	Wellinghausen et al. [7]	17/M	Sputum	Clinically stable	CF	API 20 NE (*Sphingomonas paucimobilis*) 16S rRNA sequencing (*Inquilinus limosus*) with 99.8% homology	Mucoid
**8**	Wellinghausen et al.^a^, Schmoldt et al. [7, 10]	14/F	Sputum	Clinically stable^a^, exacerbation (when I. limosus was detected a second time at the age of 15 years)	CF	API 20 NE (*Sphingomonas paucimobilis*)^a^ 16S rRNA sequencing (*Inquilinus limosus*) with 99.3% homology^a^ RAPD-PCR analysis (*Inquilinus limosus*) 16S rRNA sequencing (*Inquilinus limosus*) with 100% homology	Mucoid^a^/ extremely mucoid
**9**	Kiratisin et al. [16]	38/F	Blood cultures	Early-onset prosthetic valve endocarditis with congestive heart failure	Tetralogy of Fallot with several corrective surgery	Vitek 2 system GN (*Rhizobium radiobacter*) with 99% probability 16S rRNA sequencing (*Inquilinus limosus*) with 99.5% homology	Very mucoid
**10**	Schmoldt et al. [10]	15/F	Sputum	Severe pulmonary exacerbation^a^	CF	RAPD-PCR analysis (*Inquilinus limosus*) 16S rRNA sequencing (*Inquilinus limosus*) with 100% homology	Extremely mucoid
**11**	Schmoldt et al. [10]	18/M	Sputum	Clinically stable, serious decline 8 months later	CF	RAPD-PCR analysis (*Inquilinus limosus*) 16S rRNA sequencing (*Inquilinus limosus*) with 100% homology	Extremely mucoid
**12**	Schmoldt et al. [10]	19/F	Sputum	Exacerbation	CF	RAPD-PCR analysis (*Inquilinus limosus*) 16S rRNA sequencing (*Inquilinus limosus*) with 100% homology	Extremely mucoid
**13**	Schmoldt et al. [10]	16/F	Sputum	Clinically stable	CF	RAPD-PCR analysis (*Inquilinus limosus*) 16S rRNA sequencing (*Inquilinus limosus*) with 100% homology	Extremely mucoid
**14**	Schmoldt et al. [10]	32/M	Sputum	Respiratory decline/ no exacerbation	CF	RAPD-PCR analysis (*Inquilinus limosus*) 16S rRNA sequencing (*Inquilinus limosus*) with 100% homology	Extremely mucoid
**15**	Bittar et al. [24]	17/F	Sputum	Clinically stable	CF	API 20 NE (*results not reported*) Real-time PCR assay with Taqman probe (*Inquilinus limosus*)	not reported
**16**	Bittar et al. [24]	2/M	Sputum	Productive cough	CF	API 20 NE (*results not reported*) Real-time PCR assay with Taqman probe (*Inquilinus limosus*)	not reported
**17**	Bittar et al. [24]	21/M	Sputum	Exacerbation	CF	API 20 NE (*results not reported*) Real-time PCR assay with Taqman probe (*Inquilinus limosus*)	not reported
**18**	Bittar et al. [24]	15/M	Sputum	Fever and thoracic pain	CF	API 20 NE (*results not reported*) Real-time PCR assay with Taqman probe (*Inquilinus limosus*)	not reported
**19**	Cooke et al. [19]	9/F	Sputum	Clinically stable	CF	API 20 NE (*Agrobacterium radiobacter*) 16S rRNA sequencing (*Inquilinus limosus*) with 99% homology	Mucoid
**20**	Hayes et al. [20]	20/M	Sputum	Clinical, spirometric and radiographic decline	CF	16S rRNA sequencing (*Inquilinus limosus*)	Mucoid
**21**	Salvador-García et al. [21]	23/F	Sputum	Clinically stable	CF	API 20 NE (*Sphingomonas* *paucimobilis* or *Rhizobium radiobacter*^b^) Vitek 2 GN (*Roseomonas gilardii*) 16S rRNA sequencing (*Inquilinus limosus*) with 99.3% homology	Extremely mucoid
**22**	Cicatiello et al. [18]	20/M	Sputum	Clinically stable	CF, two episodes of acute pancreatitis in the past	MALDI-TOF (*Inquilinus limosus*) 1.697 16S rRNA sequencing (*Inquilinus limosus*) with 99.9% homology	Mucoid
**23**	Goeman et al. [22]	31/M	Blood culture, pleural fluid, empyema rind, thoracotomy wound tissue	Postoperative fever, worsening pulmonary infiltrates, in follow-up bilateral pulmonary empyema and wound dehiscence on the left	CF with bilateral lung transplantation	API 20 NE (*Sphingomonas paucimobilis*) MALDI-TOF (*Inquilinus limosus*) 1.529 Cellular fatty acid analysis (CFAA) by gas chromatography (*Inquilinus limosus*)	Highly mucoid
						16S rRNA sequencing (*Inquilinus limosus*) with 100% homology	
**24**	McHugh et al. [17]	60/F	BAL	Shortness of breath, chronic dry cough, increasing supplemental oxygen demand, multiple pulmonary radiographic pathological changes	Since 1 year recurrent episodes of bronchitis	API 20 NE (no identification) VitekMS (no identification) Biotyper (*Inquilinus limosus*) > 2.40 16S rRNA sequencing (*Inquilinus limosus*) with 99.0% homology	not reported
**25**	Poore et al. [25]	6/M	BAL	Fever, cough, chest pain, pneumonic infiltrates, respiratory decline	CF	Identification done in Reference Centre—method not reported	not reported
**26**	Watson et al. [26]	19/M	Sputum	Spirometric decline	CF	MALDI-TOF (*Inquilinus limosus*)	Not reported
**27**	Watson et al. [26]	22/M	Sputum	Spirometric decline	CF	MALDI-TOF (*Inquilinus limosus*)	Not reported
**28**	Watson et al. [26]	25/M	Sputum	Spirometric decline	CF	MALDI-TOF (*Inquilinus limosus*)	Not reported
**29**	Ríos-López et al. [23]	12/M	Sputum	Clinical, spirometric and radiographic decline	CF	API 20 NE (*Brevundimonas vesicularis*) MALDI-TOF (*Inquilinus limosus, score value* 2.47)	Mucoid
**30**	Farfour et al. [27]	45/F	Sputum, BAL, Blood culture	Clinical, Respiratory and radiological decline,	End-stage CF with Lung transplantation, SARS-CoV-2	MALDI-TOF (*Inquilinus limosus*)	Not reported

*BAL* Bronchoalveolar lavage

^a^died despite ongoing intensive therapy at the age of 16 years

^b^*Rhizobium radiobacter* is a synonym of* Agrobacterium radiobacter*

Identification methods based on the evaluation of biochemical reactions and metabolic properties (such as API 20NE and VITEK2) are not suitable for the reliable identification of *I. limosus*. In these cases, it is reported to be misidentified as *Sphingomonas paucimobilis, Pseudomonas spp., Roseomonas gilardii* or *Agrobacterium radiobacter* [7, 15, 17, 21, 22]. These limitations can be explained since corresponding entries specific for *I. limosus* are not yet available in the respective databases making itsidentification not possible [7, 22, 24].

In contrast, MALDI TOF MS seems to be a good option for species identification when corresponding spectra are available [17, 23]. There are results from a number of research groups that suggest that *I. limosus* can be reliably identified using the MALDI biotyper (Bruker Daltonics, Bremen, Germany) [17, 23, 26]. However, Cicatiello et al. reported an identification of *I. limosus* with a score value of only 1.697 [18]. Since a score below 1.7 means that the species is „not identified “, sequencing of the 16S rRNA gene was additionally carried out, which confirmed the species *I. limosus* [18, 28, 29]. Unfortunately, the authors did not go into detail about the results of the MALDI TOF MS analysis. In this case, the low score value could be explained to be due to errors in carrying out the analysis (e.g. sample application). However, from our own studies on rare human pathogens, we know that in these cases the analysis by MALDI TOF MS gives good indications of the genus present [30]. For this reason, it would be interesting to examine such an isolate more closely at the genomic level and to find out whether it could be a representative of another species of the genus *Inquilinus*. So far, only one report attempting to identify *I. limosus* using VITEK MS (bioMérieux) is available [17]. In this case, an identification of the isolate failed [17]. Therefore, one may speculate that the corresponding spectra were probably not yet available in the database at this point of time. Moreover, there are currently no reports on the suitability of MALDI TOF MS for the identification of *I. ginsengisoli*. Therefore, it is not yet possible to give a statement whether MALDI TOF MS can be used to differentiate between the two species *I. limosus* and *I. ginsengisoli.* For this reason, a comparative study on the suitablility of MALDI TOF MS in which both species are compared against each other should be a matter of future research.

Futhermore, in many reports, the statement was made that a reliable identification of *I. limosus* is also possible using the sequencing of the 16S rRNA gene [15, 17, 22]. However, since the sequences of the 16S rRNA gene of *I. limosus* and *I. ginsengisoli* are very similar (about 98% identity with the sequence of the *I. limosus* type strain), it may be difficult (despite the currently recognised cut-off of 98,7% homolgy) to reliably differentiate the two species [3, 16, 31]. For this reason, one can only reliably confirm the genus Inquilinus using 16S rRNA sequencing [16]. In order to achieve a secure distinction of the two species, Jung et al. therefore suggest to carry out a DNA-DNA hybridisation, which however is only feasible for research and not for routine clinical diagnostics purposes due to the considerable personnel and methodological effort needed [3]. Based on own experiences with the identification of rare human pathogenic bacteria however, the preparation of the whole genome and subsequent determination of the species with the digital DNA-DNA hybridisation (dDHH) seems to be the most suitable procedure to securely clarify the species [30, 32]. Unfortunately, this method is currently not yet suitable for routine use and is therefore reserved mainly for research purposes. An overview of the experiences with the identification of *I. limosus* to date is summarised in Table 3.

### Microbiology

The pathogenesis of *I. limosus* infections is not yet well understood. An important observation is that almost all isolates reported by now were derived from clinical samples of CF patients. This disease has its aetiology in mutations of the transmembrane conductance regulator (CTFR). The CTFR receptor plays a crucial role in the mucociliary clearance hence, its distortion leads to an increased mucus plug formation [17]. Additional factors, which are associated with CF such as increasing hypoxia and impaired neutrophil functions, predisposes the airways of CF patients to chronic respiratory infections as well [33]. In this way a microenviroment is created, that promotes bacterial growth and consequently favours the development of chronic respiratory infections. In another case, however, *I. limosus* was detected in a 60-year-old patient with acutely developing bronchitis who did not suffer from CF [17]. Diseases of the upper respiratory tract or the lungs could therefore be regarded as a general risk factor for *I. limosus* colonisation or even a precondition for the emergence of an infection. However, there was another case report, that deals with an *I. limosus*-associated early-onset prosthetic valve endocarditis in a tetralogy of Fallot patient. No additional lung disease was reported in this case. Here, *I. limosus* was isolated from blood cultures [16]. Unfortunately, no information was provided about conceivable or potential risk constellations such as the status of the patient`s immune system or presence of other respiratory tract anomalies in the patient.

The mode of transmission of *I. limosus* is also unsolved. Evidence of transmission however are provided by Watson et al., who reported *I. limosus* infections in three co-habiting brothers with CF [26]. Here the authors suggested either inter-sibling transmission or simultaneous acquisition from a common source, as the reason for the infections in the three brothers [26]. Nosocomial *I. limosus* acquisition has also been suggested as a likely source in two patients [16, 24]. Wellinghausen et al. suggested environmental sources as a potential reservoir of *I. limosus* due to its relation to other nonfermentative bacteria [7]. In a recent study, *Inquilinus limosus* was identified as one of the commensals from the oral cavity of a domestic cat [8]. This raises the possibility of a zoonotic transmission. However, further studies are needed in analysing the environmental stability and transmission of *I. limosus* in order to fully understand how and whether transmission from the environment or between humans or from animals to humans could be possible. Future case reports on *Inquilinus limosus* could also report on the presence of domestic pets in the homes of the patients. This information could be helpful in identifying possible sources or reservoir of the bacteria.

In most cases involving *I. limosus* infections, a mucoid morphology of the strains was reported [7, 15, 16, 19, 20, 22]. The importance of this colony type could assume a similar significance in respiratory infections as has already been described for *P. aeruginosa*. The mucoidal appearance of *P. aeruginosa* is known to be due to profuse production and secretion of the exopolysaccharide (EPS) alginate [34]. Alginate has been postulated to act as a direct barrier against phagocytic cells and thus effectively prevent bacterial opsonization. Furthermore, Alginate seems to harbour an immunomodulatory function and contributes to adhesion and antibiotic resistance [34]. Herasimenka et al. investigated the EPS produced by *I. limosus* [35]. Their results revealed that *I. limosus* produced two mainly homopolymeric EPS that exhibited the same charge per sugar residue as alginate, which is the EPS produced in *P. aeruginosa* [35]. The similarities in the EPS of these two organisms could therefore suggest a similar mechanism. In the case of *I. limosus*, there appears to be an association between the mucoidal appearance of the bacteria and its pathogenic effects which could thus be speculated to play a role in its pathogenicity via EPS secretion. Interestingly, mucoidal forms of *P. aeruginosa* often correlate with a poorer outcome of the patient, which suggest a clinical significance of EPS as a virulence factor [34]. Therefore, it is likely that EPS of *I. limosus* could have an impact on the patient`s outcome as well. This hypothesis is strengthened by another case report. Chiron et al., isolated a non-mucoid *Inquilinus* spp. in a CF-patient. This patient showed stable clinical conditions despite not receiving specific treatment for *Inquilinus*. Afterwards, there was no further isolation of the species in this patient [15]. However, since the data in this regard are still very sparse, it is necessary to pay a particular attention in future studies whether the detection of mucoid *I. limosus* isolates could have an impact on the patient`s outcome as well.

In a study to investigate the role of emerging CF-bacterial species in biofilm development, it was shown that *Inquilinus* spp. was able to adapt and thus survive in variable-oxygen atmospheres as they exist in biofilms [23, 36]. Futhermore, it could be shown that there were hardly any differences in the growth dynamics under microaerophilic and anaerobic atmospheres. This in turn suggests that these bacteria are capable to survive under the atmospheric conditions of a biofilm. Phenotypic switching to adapt to anaerobic conditions is known to occur in organisms like *P. aeruginosa*, which is also a common CF-airway colonizer [37]. In another study to observe the behaviour of *I. limosus* with bronchial epithelial cells, Lamberti et al. found out that the bacteria invaded the epithelial cells, residing and even surviving in acidic phagosome environment albeit with slower growth rates (acid tolerance response) [38]. Furthermore, the authors found out that *I. limosus* invading the bronchial epithelial cells failed to induce IL-6 and IL-8, which are important markers of pro-inflammatory response [38]. This further suggests the ability of the bacteria to withstand or adapt to difficult growth environments while also evading the cell defense mechanisms. From these studies, it could thus be speculated that *I. limosus* chronically colonizes the respiratory epithelial cells of CF patients by either growing as biofilms along the epithelial cells or by invading the epthelial cells to reside in phagosomes. In both instances, the bacteria was able to withstand and survive in difficult and changing physiologic and biochemical conditions of the respiratory tract. However, it remains unclear how or if *Inquilinus* surviving in such harsh conditions is still capable of causing a clinical infection on its own.

The isolation of *I. limosus* from clinical specimens is often associated with co-culture of other pathogens such as *P. aeruginosa* [7, 10, 15, 23, 25]. In a recent study, it was shown that a co-infection/co-colonization of *P. aeruginosa* and *I. limosus* led to a substantial increase in the induction of neutrophil extracellular traps (NETs) as compared to either *P. aeruginosa or I. limosus* growing alone [23]. NETs are mechanisms by which activated neutrophils undergo various morphological changes leading to the release of their DNA and other proteolytic substances that helps to entrap, exterminate and prevent the spread of pathogens [39]. However, the excessive production of NETs promotes sputum viscosity, allowing for the colonization of bacteria und consequently reducing the respiratory capacity [40]. Thus, one can infere that the co-infection/co-colonization of *P. aeruginosa* and *I. limosus* would lead to an increased production of NETs which in turn contributes to further bacteria colonization in the CF patients airways, ultimately leading to disease exacerbation. What remains however unclear is if a co-infection of *I. limosus* and other organisms apart from *P. aeruginosa* also leads to substantial NETs production. One can however speculate that the substantial induction of NETs upon *I. limosus* co-infection with *P. aeruginosa*, could provide the spark that fosters the transition from *Inquilinus* colonization to *Inquilinus* infection. This speculation is supported by clinical evidence that patients’ clinical conditions worsened once *I. limosus* is detected as part of a polymicrobial spectrum in clinical specimens. In these cases, lung functions declined and only resolved after aggressive treatment was directed at *I. limosus* (Table 4). Other pathogens that have been co-cultured with *I. limosus* from the documented cases include *Staphylococcus aureus, Stenotrophomonas maltophilia, Achromobacter xylosoxidans*, Candida and Aspergillus species and non-tuberculous mycobacterium [7, 10, 15, 17, 22, 25–27]. In one case, SARS-CoV-2 Infection was detected during an *I. limosus* infection [27]. However, these other non-*P. aeruginosa* pathogens were only detected sporadically and not as often as the detection of *P. aeruginosa*. However, it is currently unknown if the co-infection of *I. limosus* with these other pathogens also induce substantial NETs production like *P. aeruginosa* does*.* Future studies can therefore look to investigate the induction of NETs formation during co-infection/co-colonization of *I. limosus* with other non-*P. aeruginosa* pathogens.

**Table 4:** Impact of *I. limosus* infections on lung function parameters

References	FEV1 (% predicted) before* I. limosus* detection	FEV1 (% predicted) after *I. limosus* detection	FEV1 (% predicted) after specific *I. limosus* treatment
Chiron et al. [15]	NG^a^	63	73
Chiron et al. [15]	NG^a^	93	94
Chiron et al. [15]	NG^a^	51	74
Chiron et al. [15]	NG^a^	120	103
Chiron et al. [15]	NG^a^	43	58
Wellinghausen et al, Schmoldt et al. [7, 10]	77	57	NG^a^
Schmoldt et al. [10]	30	24	NG^a^
Schmoldt et al. [10]	104	95/90	NG^a^
Schmoldt et al. [10]	41	26	NG^a^
Schmoldt et al. [10]	35	45	NG^a^
Schmoldt et al. [10]	122	105	NG^a^
Poore et al. [25]	87	68	81
Watson et al. [26]	60.9	46.9	64.1
Watson et al. [26]	77.4	72.9	65
Watson et al. [26]	46	43	52
Farfour et al. [27]	78	30	NG^a^

^a^*NG* Not given

Interestingly, there have also been a few *I. limosus* monoinfections [10, 15]. In the clinical cases involving *I. limosus* monoinfection, the initial mild respiratory disease, persisted and deteriorated further despite adequate antibiotic therapy, eventually leading to no clinical improvement [10, 15]. In one of the cases, a meropenem-resistant *Inquilinus* variant even emerged during antibiotic therapy [10]. Here, we speculate that the *Inquilinus* from the monoinfections could have previously existed in a polymicrobial state where they underwent treatment that was however not specific for *Inquilinus*. This would have subsequently led to the extermination of the other susceptible bacteria species and with *Inquilinus* undergoing further genotypic or phenotypic modifications that makes them even more resistant to treatment. However, there have only been two documented cases involving *I. limosus* monoinfections till date which makes it difficult to fully estimate their effects. It could also be argued that the *Inquilinus* monoinfections described above may not even necessarily be a true monoculture but rather a reflection of the limitation of the sputum culture in detecting all the pathogens present in the lower respiratory tract. A bronchioalveolar lavage analysis for example, might have been helpful here in truly estimating the bacteria populations that are involved in the disease. The studies above on *Inquilinus* monoinfections however suggest that a monoculture of *I. limosus* either alone or after previously existing in a polymicrobial state, could denote a poor patient outcome. Altogether, these clinical cases suggest the pathogenic potential of *I. limosus* either individually or in combination with other pathogens.

In recent years, CFTR modulator therapies have significantly impacted the landscape in the management and treatment of CF patients [41, 42]. These small molecule drugs aim to partially correct and improve the function of mutant CFTR proteins [43, 44]. The restoration of CFTR Function in turn has been associated with improved airway surface hydration and mucociliary clearance, increased airway surface fluid pH and reduced static airway mucus [41, 42, 45]. These effects in turn are expected to lead to reduced bacterial burden of CF pathogens such *P. aeruginosa* in the CF lungs [45]. Having discussed the potential role of *P. aeruginosa* in the pathogenicity of *I. limosus*, it can thus be further speculated that the advent of CFTR modulator therapies might lead to a reduced detection of *I. limosus* infections in the future. Future studies into the prevalence and clinical significance of *I. limosus* since the advent of CFTR modulator therapies would help to provide more insights to this speculation.

*Inquilinus* limosus has been shown from different studies to be very resistant to various antimicrobial agents [5, 15, 18, 20, 23]. The antibiotic susceptibility tests from these studies were done using either broth microdilution tests, disk-diffusion tests, E-tests or their combinations. From the studies, the bacteria were typically sensitive to Imipenem, Meropenem and Ciprofloxacin while showing total resistance to Penicillins (± beta-lactam inhibitors), Cephalosporines and Colistin. Variable resistance to the aminoglycosides was also observed [5, 15, 18, 20, 23]. A comparison is shown in Table 5.

**Table 5:** Overview of published antimicrobial susceptibilities of *Inquilinus limosus*

MIC (µg/ml)/ Disk-diffusion/E-test (µg/ml)								
**Antimicrobial agents**	Pitulle et al. [5]	Chiron et al. [15]	Wellinghausen et al. [7]	Hayes et al. [20]	Cicatiello et al. [18]	McHugh et al. [17]	Ríos-Lopez et al. [23]	Lenhart-Pendergrass et al. [46]
Ampicillin-sulbactam	> 32^a^	NT	NT	NT	R^b^	> 16^a^	> 16^c^	NT
Piperacillin-tazobactam	> 128^a^	(R)^b^	(> 256)^c^	(> 256)^c^	R^b^	> 128^a^	> 64^c^	0%^d^
Cefotaxime	> 32^a^	(R)^b^	(> 32)^c^	NT	R^b^	NT	> 32^c^	NT
Ceftazidime	> 32^a^	(R)^b^	(0.5—> 32)^c^	(> 256)^c^	R^b^	NT	> 16^c^	0%^d^
Cefepime	> 32^a^	(R)^b^	NT	(> 256)^c^	R^b^	> 32^a^	> 16^c^	0%^d^
Imipenem	2^a^	(S)^b^	(0.006–0.016)^c^	NT	0.094^c^	NT	1^c^	100%^d^
Meropenem	2^a^	NT	NT	(0.19–24)^c^	0.094^c^	8^a^	1^c^	96%^d^
Ciprofloxacin	4^a^	(S)^b^	(0.032–0.064)^c^	(0.5–24)^c^	0.064^c^	1^a^	1^c^	53%^d^
Levofloxacin	4^a^	NT	NT	NT	NT	NT	2^c^	64%^d^
Amikacin	32^a^	(I-R)^b^	(8—> 256)^c^	(32- 64)^c^	R^b^	32^a^	16^c^	83%^d^
Tobramycin	R^b^	(R)^b^	(> 256)^c^	(16–24)^c^	NT	> 8^a^	> 8^c^	4%^d^
Gentamicin	R^b^	(S-R)^b^	(8—> 256)^c^	NT	R^b^	> 8^a^	> 8^c^	50%^d^
Polymyxin B	R^b^	NT	NT	(> 1024)^c^	NT	NT	NT	NT
Colistin	R^b^	(R)^b^	(R)^b^	(> 256)^c^	R^b^	NT	NT	25%^d^
Rifampin	NT	(S-R)^b^	NT	NT	NT	NT	NT	NT
Aztreonam	NT	R^b^	NT	> 256^c^	R^b^	NT	R^b^	0%^d^
Doxycycline	16^c^	R^b^	NT	NT	NT	NT	NT	50%^d^
Minocycline	NT	NT	NT	NT	NT	NT	NT	60%d
Trimetoprim-Sulfamethoxazole	NT	R^b^	> 32^c^	NT	> 32^c^	> 4^a^	S^b^	27%d
Ticarcillin-Clavulanate	> 128^a^	R^b^	NT	NT	NT	NT	NT	0%d

*S* Sensitive, *I* Intermediate, *R* Resistant, *S-R* from Sensitive to Resistant, *I-R* from Intermediate to Resistant, *NT* Not Tested in study, MIC- Minimal Inhibitory Concentration

Results in brackets represent the value range when more than 1 antibiotic susceptibility test was done

^a^Broth microdilution (µg/ml)

^b^Disk-diffusion

^c^E-test values (µg/ml)

^d^results reported as percentage (%) Susceptibility (method used not given)

Pino et al. identified multiple chromosomal-encoded multidrug resistance efflux pumps such as the *cmeABC* operon, seven penicillin-binding proteins and four putative beta-lactamase genes from *I. limosus* isolates which could help to explain their intrinsic resistance mechanisms [47]. One of the chromosome-encoded class C beta-lactamases has been described as INQ-1 [48]. INQ-1 was suggested to be a cephalosporinase compatible with beta-lactamases belonging to group 1 of the functional classification scheme [48]. The defining characteristics of group 1 beta-lactamases are their ability to cause marked hydrolysis of cephalosporins and cephamycins while being resistant to inhibition by clavulanic acid, tazobactam or EDTA [49]. INQ-1 could explain the resistance to penicillins and cephalosporines in *Inquilinus* spp. Cases of meropenem and ciprofloxacin resistant *I. limosus* infections have likewise been described [10, 36]. Chiron et al. also described the failure of *Inquilinus* eradication with Imipenem therapy in CF patients despite in vitro susceptibility [15]. However, it remains unclear if other resistance mechanisms were in place in these resistant cases. Studies have shown that acquisition of other resistance mutations in group 1 beta-lactamases producing organisms might even lead to an extended, clinically significant resistance to antimicrobial agents [49, 50].

Lopes et al. found out that *Inquilinus* growing as biofilms were highly recalcitrant to most antibiotics tested whereas the planktonic *Inquilinus* cells were in most cases susceptible to the same antibiotics. In addition, the minimum biofilm eradication concentrations (MBEC) were much higher than the minimum inhibitory concentrations (MIC) for the planktonic population [51]. In practice, the MIC from the planktonic bacteria cell populations are usually used for the antibiotic susceptibility testing. Perhaps, testing for the MBEC of *Inquilinus* with their biofilm populations could help to prevent therapy resistance. However, it remains unclear if such high antibiotic doses are needed for the MBEC can also be applied in vivo without causing significant side-effects.

**Fig. 1:**
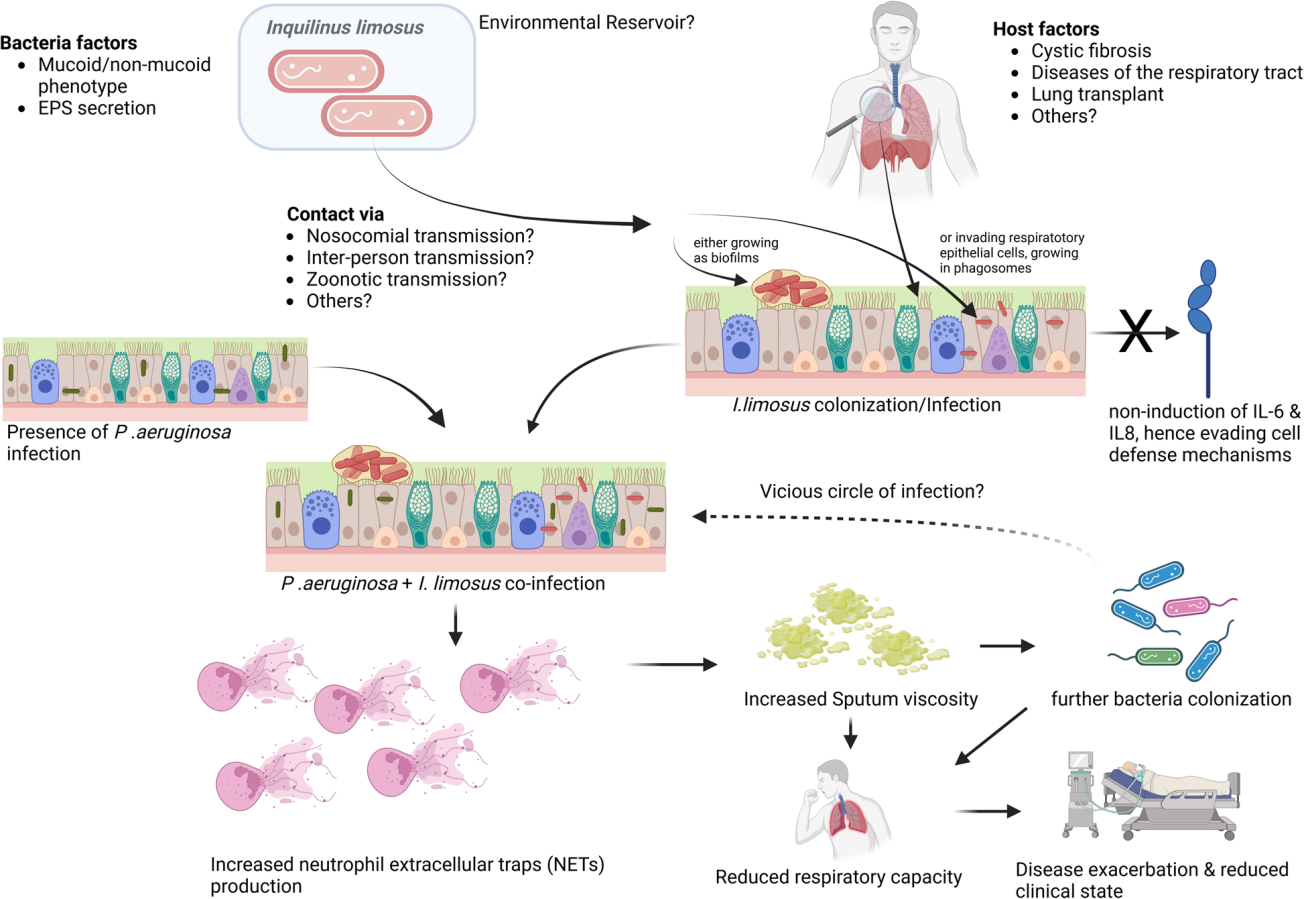
Current concept of the pathogenesis of Inquilinus limosus infections

In summary, the mucoidal nature of *I. limosus*, its ability to form biofilms and/or invade bronchial epithelial cells and to survive in variable-oxygen and pH atmospheres and its ability to produce EPS and as well as its substantial induction of NETs via co-infection with *P. aeruginosa* are potential mechanisms by which the bacteria colonizes CF airways and exerts its pathogenicity (Fig. 1). Future research into the structural and chemical components of the mucoidal layer and biofilms of *Inquilinus* might provide further insights into their pathogenic mechanisms.

### Clinical aspects

The majority of *I. limosus* airway colonisations and/or infections have been documented in CF patients [5, 7, 15]. The clinical presentation of these patients ranges from „stable “ conditions (without symptoms) to severe respiratory exacerbations, characterised by severe pneumonia, lung functions decline and respiratory failure [9, 10, 15, 17]. The respiratory exacerbations caused by *I. limosus* infections have been estimated to occur at rates similar to chronic *Pseudomonas aeruginosa* infections in CF patients [46]. Table 3 provides an overview of the clinical features of infections and chronic colonizations caused by *Inquilinus*. Differentiating between *Inquilinus* airway colonization and *Inquilinus* infection is difficult considering the paucity of published data on the subject. Most probably, *I. limosus* airway colonization presents asymptomatically with clinically stable conditions while *I. limosus* infections lead to varying clinical symptoms. However, it is unclear when or how a transition from chronic *I. limosus* colonization to *Inquilinus* infection occurs as well as the factors that influence such transitions. Perhaps, an understanding of the pathogenicity of *Inquilinus* might offer some insights on this issue. In addition to causing pulmonary disease, *I. limosus* associated bacteremia, empyema, wound infection and endocarditis have also been described in literature [16, 22, 27]. Due to its increasing clinical significance, *I. limosus* has often been described as an emerging pathogen of interest in CF patients [25]. Treating *I. limosus* infections is usually challenging considering the difficulties with the identification, the multi-resistant nature and the relative unclear pathogenicity of the bacteria. The first step to a successful therapy is the identification and isolation of *Inquilinus* in culture. Due to the bacteria slow-growing nature, it might be necessary to incubate culture media-containing patient’s materials for a longer time (up to 72 h). Detailed information about the patient’s signs and symptoms can also be given to the diagnostic laboratory to aid with the identification. This might particularly be helpful for example, in cases of recurring pulmonary disease or exacerbations in CF patients despite antibiotic therapy. Following identification, *I. limosus* infections have been successfully treated with a high dose of the susceptible antibiotic agent per in vitro antibiotic susceptibility tests (Table 5). The length of the therapy from successful treatments usually varies typically from a minimum of two weeks to a maximum of six weeks (Table 6). If necessary, other supportive therapy such as supplementary oxygen can be evaluated. Further patient samples can also be obtained and sent to the diagnostic laboratory to observe changes in bacteria growth dynamics under therapy and with antibiotic susceptibility tests. Finally, an interdisciplinary approach with physicians, microbiologists and infectious disease specialists should be encouraged in the management of *I. limosus* infections.

**Table 6:** Selected examples of therapy modalities from successful treatment of Inquilinus infections

	**Therapy modalities before Inquilinus detection**	**Therapy modalities after Inquilinus detection**	**Clinical response/Effects**	**Reference**
1	Ceftriaxone and trimethoprim-sulfamethoxazole	4 weeks course of intravenous meropenem, amikacin and oral ciprofloxacin	Clinical and respiratory improvements	Poore et al. [25]
2	Linezolid, clofazimine and voriconazole	14 days ciprofloxacin	Clinical stabilization	McHugh et al. [17]
3	cefepime ciprofloxacin, cotrimoxazole, antiviral and antifungal agents	6 weeks course of intravenous meropenem andgentamicin	Recurring pleural effusions followed by clinical improvment; intermittent isolation of *I. limosus* over 11 years	Goeman et al. [22]
4	Not reported	14 days course of intravenous amikacin and oral ciprofoxacin	Clinical improvement, chronic colonization of mucoid *I. limosus*	Hayes et al. [20]
5	Vancomycin Rifampin Gentamicin and Ceftriaxone	6 weeks course of Meropenem	Recovered with clinical and cardiac improvement	Kiratisin et al. [16]

### Summary

*I. limosus* represents underestimated bacteria that contributes to CF airway colonization and/or infection with potentially severe clinical implications. Increasing number of case reports has led to a better understanding of the clinical significance of *I. limosus* infections and the underlying pathomechanisms, particularly in CF patients. The natural habitat of *Inquilinus* is still unknown however environmental sources have been suggested to be a likely reservoir of this bacteria. The microbiological identification of *Inquilinus* is difficult due to its slow-growing nature and due to the absence of the bacteria in the databases of most commercial identification systems. Furthermore, a reliable differentiation of *I. limosus* from other species of the genus is still not possible with the sequencing of the 16S rRNA gene. Additionally, a comparative study with a defined collection of isolates on the suitability of MALDI TOF MS for the identification of *I. limosus* is also currently lacking. Therefore, a general recommendation regarding the most suitable routine method for species identification can not be proposed yet.

The mucoidal nature of *I. limosus* and its ability to produce EPS, the ability to form biofilms and/or invade bronchial epithelial cells and to survive in variable-oxygen and pH atmospheres as well as its substantial induction of NETs via co-infection with *P. aeruginosa* are potential mechanisms by which *Inquilinus* exerts its pathogenic effects. The clinical presentation of *Inquilinus* infections ranges from asymptomatic to severe respiratory exacerbations, characterised by severe pneumonia, lung functions decline and respiratory failure. *I. limosus* shows intrinisic resistance to multiple antimicrobial agents including penicillins (± beta-lactam inhibitors), Cephalosporines and Colistin. Successful treatments of *I. limosus* infections often involves high doses of susceptible antibiotics administered over 2 to 6 weeks, combined with measures of supportive therapy.

## Data Availability

No original data are analysed in this manuscript. The genome data mentioned in this manuscript are available via NCBI.
